# Vestibular Testing—New Physiological Results for the Optimization of Clinical VEMP Stimuli

**DOI:** 10.3390/audiolres13060079

**Published:** 2023-11-09

**Authors:** Christopher J. Pastras, Ian S. Curthoys

**Affiliations:** 1 Faculty of Science and Engineering, School of Engineering, Macquarie University, Sydney, NSW 2109, Australia; christopher.pastras@mq.edu.au; 2 Vestibular Research Laboratory, School of Psychology, The University of Sydney, Sydney, NSW 2006, Australia

**Keywords:** VEMP, otoliths, compound action potential, CNQX, non-quantal, resistive coupling, sound, vibration

## Abstract

Both auditory and vestibular primary afferent neurons can be activated by sound and vibration. This review relates the differences between them to the different receptor/synaptic mechanisms of the two systems, as shown by indicators of peripheral function—cochlear and vestibular compound action potentials (cCAPs and vCAPs)—to click stimulation as recorded in animal studies. Sound- and vibration-sensitive type 1 receptors at the striola of the utricular macula are enveloped by the unique calyx afferent ending, which has three modes of synaptic transmission. Glutamate is the transmitter for both cochlear and vestibular primary afferents; however, blocking glutamate transmission has very little effect on vCAPs but greatly reduces cCAPs. We suggest that the ultrafast non-quantal synaptic mechanism called resistive coupling is the cause of the short latency vestibular afferent responses and related results—failure of transmitter blockade, masking, and temporal precision. This “ultrafast” non-quantal transmission is effectively electrical coupling that is dependent on the membrane potentials of the calyx and the type 1 receptor. The major clinical implication is that decreasing stimulus rise time increases vCAP response, corresponding to the increased VEMP response in human subjects. Short rise times are optimal in human clinical VEMP testing, whereas long rise times are mandatory for audiometric threshold testing.

## 1. Introduction

The classical methods of testing human otolith function using linear acceleration stimuli have now been complemented in the clinic by the use of air-conducted sound (ACS) and bone-conducted vibration (BCV) as vestibular, and specifically otolithic, stimuli [1,2,3]. Sound and vibration are more clinically practical, and physiological and anatomical evidence shows that the otolith organs are activated by such stimuli (for reviews, see [4,5]).

**Figure 1:**
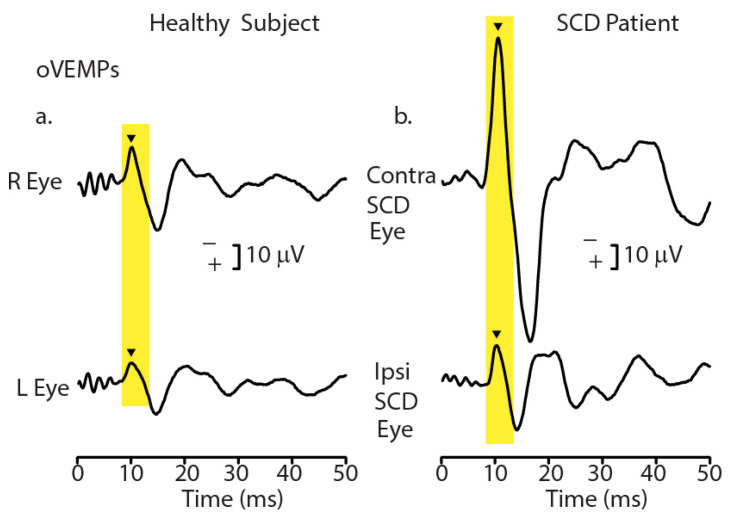
Recordings of ocular vestibular evoked myogenic potentials (oVEMPs) to 500 Hz bone-conducted vibration (BCV) from a healthy subject (**a**) and a patient with a CT-verified semicircular canal dehiscence (SCD) (**b**). In each record, the stimulus onset occurred at time 0. In the healthy subject, BCV at the midline of the forehead at the hairline (Fz) causes symmetric myogenic potentials (oVEMPs) recorded by surface electrodes beneath the eyes as the patient looks up, with approximately equal amplitude oVEMP n10 potentials (arrowheads). In contrast, the same Fz stimulus causes asymmetric n10 potentials of the oVEMP response in the SCD patient; the oVEMP n10 recorded from beneath the contralesional eye is much larger than the oVEMP n10 recorded from beneath the ipsilesional eye, indicating a stronger response from the affected ear (with the SCD). Redrawn with permission from the authors of [10].

ACS or BCV stimuli can activate vestibular receptors, resulting in small myogenic potentials called vestibular evoked myogenic potentials (VEMPs). VEMPs are due to synchronized action potentials of vestibular afferents activated at the onset of the stimulus since, as we explain below, prolonging the stimulus rise time degrades the synchronization of action potentials and degrades VEMPs [6]. VEMPs are being used to identify unilateral utricular and saccular loss [7,8,9], semicircular canal dehiscence (see Figure 1) [10], and central deficits [11].

The use of VEMPs as indicators of vestibular function rests on physiological evidence that vestibular receptors and neurons are activated by sound and vibration. Mostly, this has come from recording the response of single identified otolithic afferent neurons to sound or vibration in guinea pigs and other species (for reviews, see [4]). As we show below, new physiological results are clarifying how initial neural activation by BCV or ACS takes place, thereby validating the use of particular stimuli for clinical VEMP testing. These physiological studies use procedures that allow for the differentiation of vestibular responses from cochlear responses, since obviously both cochlear and vestibular receptors and afferent neurons are activated by ACS and BCV. Here, we review those procedures, their results, and their interpretation.

## 2. Physiological Evidence—Overview

In anesthetized guinea pigs, we have measured VIII nerve activation (mainly the compound action potential (CAP)) in response to transient ACS and BCV stimuli (usually click stimuli) [12,13,14]. CAPs are mass neural potentials caused by synchronized action potentials of many individual primary afferent neurons triggered by transient ACS or BCV stimuli such as clicks [15]. An electrode near the VIII nerve records a response composed of many electrophysiological potentials caused by ACS or BCV stimulation: among them: cochlear compound action potential (cCAP), vestibular compound action potential (vCAP), cochlear and vestibular microphonics (CM and VM) and auditory and vestibular nerve neurophonics (ANN and VNN) [16,17]. The largest component is the cochlear compound action potential (cCAP) and for convenience and to relate these data to previous results, we refer to the whole VIII nerve compound action potential as cCAP, recognizing the existence of many components in this potential. We clarify these matters below. These peripheral CAP responses are also the basis for the vestibular evoked potential (VsEP) recorded by scalp electrodes [18,19,20,21,22,23]. The VsEP is a smaller far field correlate of the localized near field response. Our experiments compare vestibular CAPs (vCAPs) to cochlear CAPS (cCAPS) under controlled experimental conditions, and that is the main focus of this review. As we show below, one particularly useful aspect of these physiological recordings is that they provide an objective measure at the vestibular periphery of the optimum stimulus parameters for recording VEMPs in the clinic.

### 2.1. Independence of Cochlear and Vestibular Labyrinthine Divisions

The vestibular and cochlear divisions of the labyrinth share the same fluids and have receptor cells of superficially similar appearance, so the vestibular and cochlear divisions appear to be interdependent. That appearance is false [24]. It has been shown that the vestibular system can function independently of the cochlea in humans and guinea pigs. Plontke et al. showed that after the total surgical ablation of the cochlea in (rare) human patients with intracochlear schwannoma, the semicircular canals and the otoliths continue to function normally [25]. In 27 consecutive patients, there was no significant decline in any of the measured indicators of canal or otolith function after total surgical cochlear ablation. This result shows conclusively that in humans, the canals and otoliths can continue to function normally after the surgical ablation of the cochlea, and as we show below, this is also the case in guinea pigs. We measured CAPs in guinea pigs after chemical cochlear ablation via potassium chloride (KCl) infusion, which progressively inactivates cochlear receptor and afferent neuron activity by changing the membrane potential of receptors and afferent neurons [26]. Tasaki and Fernandez found that localized potassium infusion was very convenient for changing or totally suppressing electrical signs of cochlear activity in a restricted region by changing the membrane potential of receptors and afferents. We refer to the compound action potential that remains after the ablation of the cochlear contribution as the vestibular compound action potential (vCAP). Its continued presence after cochlear ablation conclusively identifies the vCAP as being of vestibular origin since cochlear function is absent. The vCAP allows us to clinically measure relevant vestibular aspects of transient stimuli that activate vestibular receptors and therefore optimize the stimulus parameters, maximizing the vestibular component of VEMPs.

### 2.2. Differentiating Vestibular and Cochlear Compound Action Potentials

There are two approaches to differentiating vestibular from cochlear responses in animal experimental studies. One can either

(a) use stimuli that are accepted as otolithic—transient linear accelerations—and interpret the results (such as the VsEP) as being due to vestibular rather than cochlear activation [20]

or

(b) use transient BCV or ACS clicks, which can activate all labyrinthine sensory regions but use various controls, such as disabling or even removing the cochlea, to identify the vestibular component of the total CAP nerve response [14,26].

The problem with using linear acceleration is that the presumably specific otolithic stimulus most likely has aspects that affect the cochlea—it is difficult to deliver a transient linear acceleration without sound or vibration—so it is wise to use broadband masking noise to minimize any possible cochlear contribution [27]. As we show below, masking by broadband noise has a surprisingly little effect on vestibular receptors and afferents. To address these matters, we first briefly review the relevant anatomical and physiological evidence before showing how these physiological results relate to clinical VEMP testing.

## 3. Anatomy

### 3.1. Cochlea

Inner hair cells have a fairly uniform appearance throughout the cochlea, although stereocilia height varies along the basilar membrane (see [28,29] for reviews). In the cochlea, each individual afferent neuron contacts just one inner hair cell with a bouton synapse (see [24] for a review), but each inner hair cell receives many afferent bouton contacts from different axons. For every synapse, the transmitter is glutamate, released by synaptic ribbons in the inner hair cells [24,30,31,32]. Ribbons appear to be the main mechanism of glutamatergic synaptic transmission for cochlear primary afferents and are thought to be responsible for the temporal precision of cochlear responses to transient stimuli and the phase locking of cochlear afferent action potentials to sinusoidal stimuli [24,33,34,35].

Transient stimuli such as clicks deflect cochlear receptor hair bundles, causing the mechanoelectrical transduction (MET) channels on the stereocilia to open and depolarize the hair cell and therefore generate a receptor potential—the cochlear microphonic (CM)—and action potentials in individual afferents (see [24,29,36] for reviews). The synchronized action potentials of many simultaneously activated cochlear afferents in response to a broadband click stimulus are the major components of the mass neural response—the cochlear action potential (cCAP)—recorded by gross electrodes on, or close to, the auditory nerve. Individual primary cochlear afferents have remarkable temporal precision, as shown by the precision of their phase-locked response to sinusoidal stimuli [35]. In individual neurons, action potentials are generated at a particular stimulus phase angle (or a narrow band of phase angles) up to high frequencies (>1000 Hz) [33,35]. Classic examples of phase locking individual primary cochlear primary afferents in guinea pigs were published by Palmer and Russell in 1986 [37].

### 3.2. Vestibular

**Figure 2:**
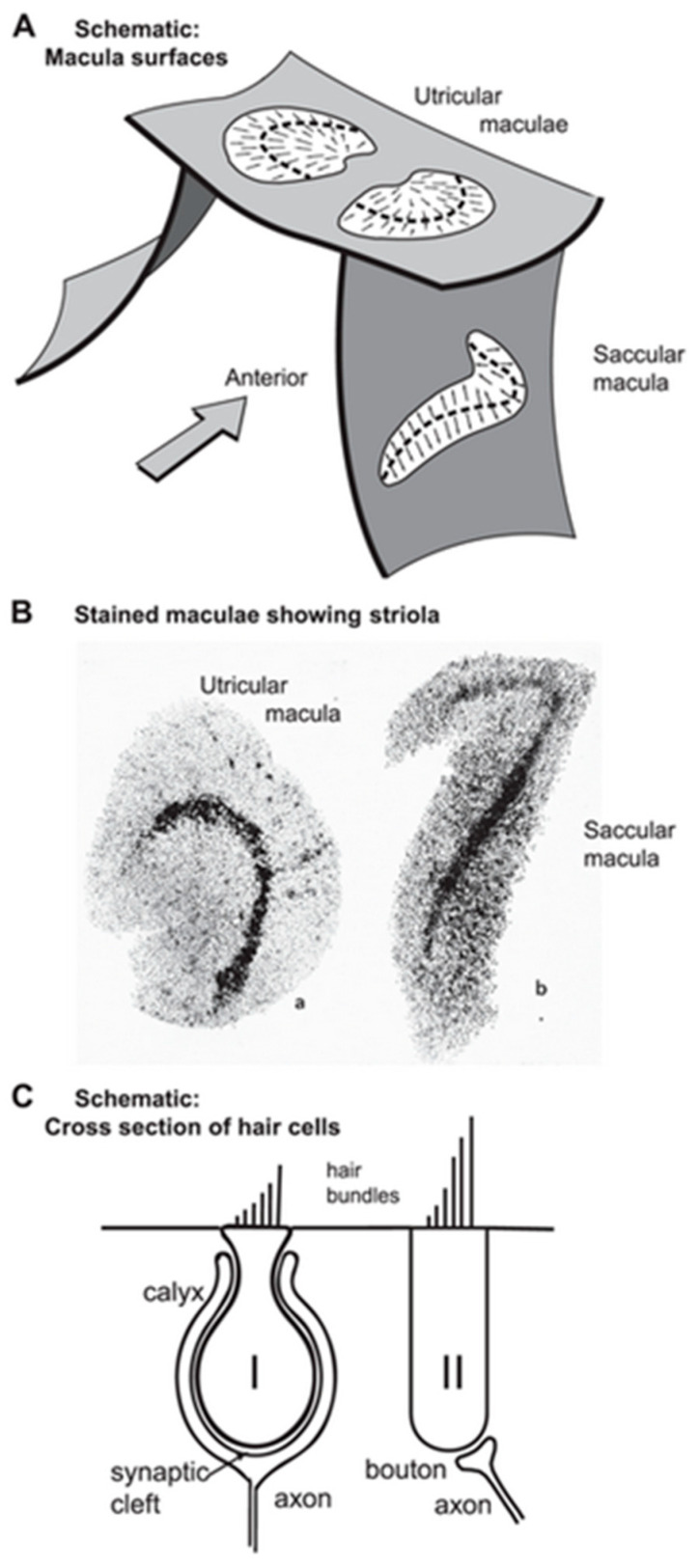
(**A**) Schematic representation of the approximate spatial relationship of the utricular and saccular maculae. The small arrows show the preferred polarization of hair cells across the maculae. The “dividing line” between oppositely polarized receptors is called the line of polarity reversal, and a band of hair cells on either side of that line is referred to as the striola (Lindeman 1969). (**B**) Views of the entire surface of a guinea pig utricular and a saccular macula following treatment using silver nitrate and succinic dehydrogenase, which stain the type I hair cells preferentially [58]. The bands of dark dots show the striola of both maculae, with a higher density of type I hair cells. (**C**) Schematic representation of type I and type II hair cells and their calyx and bouton afferent terminals, respectively. (**A**,**C**) Reproduced with permission from *Frontiers in Neurology*, ©Curthoys et al. (2018). (**B**) Reprinted from [58] with permission from Tohoku University Medical Press.

Vestibular receptors have a similar hair bundle appearance to cochlear receptors (see [29]), but there are systematic anatomical and physiological differences in vestibular receptors across vestibular receptor surfaces [38,39,40,41]. The receptors and afferents from a particular region in each otolithic macula, a stripe called the striola (see Figure 2A,B), are distinct; it is here that amphora-shaped type I receptors, enveloped by a calyx afferent ending, are plentiful (Figure 2C) [32,42,43,44,45,46]. Paralleling cochlear responses, transient vestibular stimuli such as abrupt linear accelerations or ACS or BCV clicks deflect vestibular hair bundles, causing MET channels in the stereocilia to open [28,47], resulting in a receptor potential—the vestibular microphonic (VM) [48]—and in action potentials in primary vestibular afferents. Clicks generate vCAPs, with the main contributor to the vCAP to click stimuli being from otolithic afferents with irregular resting discharge originating from type I receptors at the striola since it is these otolithic receptors and afferent neurons which are selectively activated by sound or vibration in the guinea pig [4,49,50]. Vestibular afferents with regular resting discharge synapse on type I and II receptors in extrastriolar regions [51] and have a poor or non-existent response to ACS or BCV [41,49,50,52]. Regular afferents are rarely (and then only weakly) activated by sound and vibration at clinically appropriate stimulus levels. In stark contrast, irregular otolithic afferents are activated and phase locked at low intensities to these stimuli up to high frequencies (>2000 Hz) [53,54]. The vestibular type I/calyx afferent synapse is very unusual—the calyx ending of the vestibular afferent fiber envelops the amphora-shaped type I receptor (Figure 2C) instead of making a punctate synapse as in the cochlea inner hair cells [46,55,56]. However, at this type I/calyx afferent junction, again, the transmitter is glutamate, as in the cochlea.

**Figure 3:**
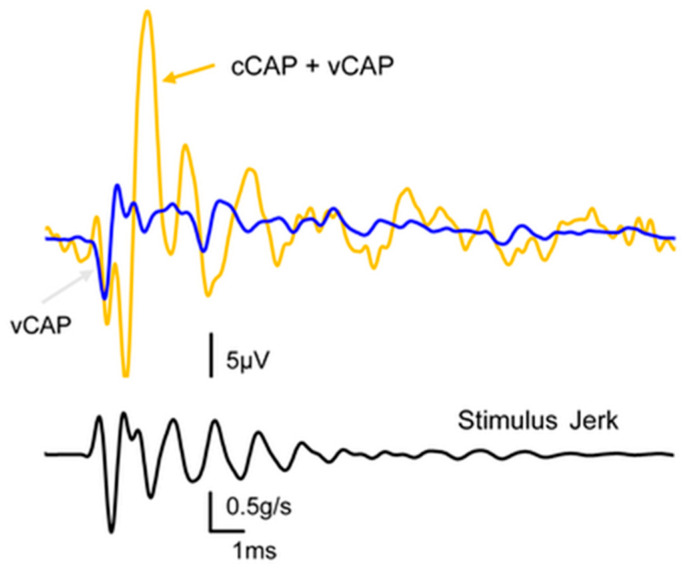
The effect of reducing cochlear contribution to the total CAP via potassium chloride infusion. The orange trace shows the compound action potential of the VIII nerve to a click stimulus, and in our terms, this shows the combined cCAP and vCAP. The blue trace shows the same response after the cochlear contribution has been eliminated via the infusion of KCl into the cochlea, and we name the remaining response the vestibular compound action potential (vCAP). The lower record shows the jerk of the click stimulus.

The vCAP is identified as being vestibular since it is present after total cochlear ablation and disappears after vestibular endorgan ablation (Figure 3). A related potential—the vestibular evoked potential (VsEP)—is recorded at the scalp, and the VsEP is also held to reflect the synchronized activity of the vestibular receptors and afferents [16,18,20,22,57]. Our simultaneous recording of both vCAP and VsEP in guinea pigs’ responses to the same stimuli shows that tight relationship, although the VsEP is smaller than the vCAP by a factor of about 100.

### 3.3. Specificity

After cochlear ablation, the cCAP is abolished, and the remaining CAP is termed the vCAP since it is generated by the remaining vestibular afferents [59] (see Figure 3). This is an extremely important control which differentiates the vCAP from the cCAP and allows us to identify optimum vestibular stimuli after eliminating cochlear contributions. Total surgical cochlear ablation involves opening the bony wall of the cochlea and removing the entire cochlear division of the labyrinth whilst leaving the vestibular division intact. In guinea pigs, the vestibular division continues to function, and transient stimuli continue to generate vCAPs after major surgery on the cochlea. The second method of cochlear ablation, chemical ablation, uses Tasaki and Fernandez’s method of infusing KCl slowly through a small hole in the bony wall of the cochlea at the apex whilst repeatedly testing the cCAP and CM to verify the decline and eventual absence of cochlear function [60].

Once cochlear function is lost, the KCl infusion is terminated and the hole is sealed so that the walls of the bony labyrinth are again intact, and transient stimuli continue to generate vCAPs.

Of the two procedures, chemical ablation is preferred since it leaves the bony wall of the labyrinth intact, whereas after the surgical ablation of the cochlea, there is a large dehiscence of the bony labyrinth, which changes the mechanical operation of the vestibular labyrinth [61] and therefore causes enhanced neural responses to ACS and BCV [62]. Another result of dehiscence is that canal afferents with irregular resting discharge, as well as the usual otolithic afferents with irregular resting discharge, will be activated by ACS and BCV stimuli [3,62]. This enhanced and broader vestibular activation is unlikely after chemical ablation by KCl since the bony wall of the labyrinth is intact. Importantly, after either cochlea ablation procedure, click stimuli can still activate vestibular receptors and generate a vCAP, confirming, in two different ways, that the vCAP is minimally contaminated by a cochlear contribution. The vCAP is probably driven primarily by otolithic afferents, most likely originating from the sound- and vibration-sensitive striolar receptors and irregular afferents, since it is these receptors and afferents which respond to ACS and BCV [49]. We contend that these different effects of cochlear vs. vestibular responses are due to the different synaptic mechanisms.

### 3.4. Transmitter

**Figure 4:**
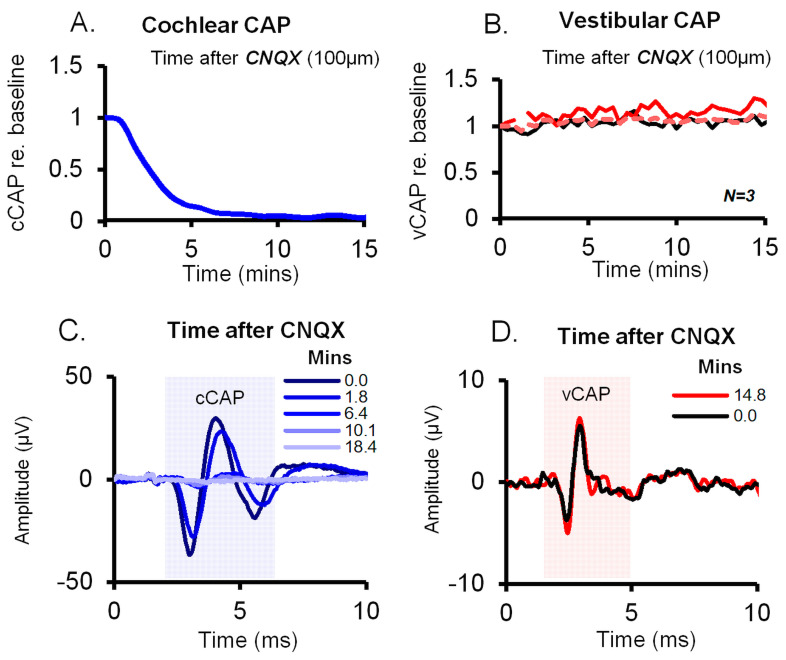
Effect of 6-cyano-7-nitroquinoxaline-2,3-dione (CNQX) on cochlear vs. vestibular CAPs in guinea pigs. CNQX (100 μM in artificial perilymph) was applied to the cochlear round window and the utricle via the macular epithelium. (**A**,**B**) Time series of cCAP and vCAP amplitudes normalized as a proportion of baseline. The vCAP baseline was after surgical cochlear removal; the cCAP was diminished after CNQX, whilst vCAPs remained unperturbed, demonstrating the different effects of CNQX on cochlear vs. vestibular afferent function. (**C**,**D**) Typical averaged cCAP and vCAP responses (100 stimulus presentations) corresponding to labeled time points after CNQX administration. CNQX blocks auditory CAPs (blue) but not the vCAP. Reproduced with permission from the authors of [14].

The neurotransmitter between receptors and primary afferents in both the cochlear and vestibular systems is the same—glutamate (see [29] for a review). However, blocking glutamate transmission via the AMPA receptor antagonist CNQX has very different effects on the two systems:

- In the cochlea, CNQX blocks cochlear afferent responses to sound—the cCAP is greatly reduced and almost completely disappears [14,24,30,31] (see Figure 4).
- In contrast, in the vestibular system, CNQX does not block vestibular activation; CNQX has very little effect on the vCAP response to BCV or ACS transient stimuli [14,48] (compare A and B in Figure 4).

Contini et al. have shown that synaptic transmission at the type I–calyx synapse is very unusual [63,64]. Contini et al. managed the remarkable feat of the simultaneous dual patch clamp recording of a type I receptor and its calyx afferent, thus providing definitive empirical evidence for non-quantal transmission and the mechanism by which it takes place. As we explain below, their results showed that there are three modes of transmission at the type I–calyx synapse. Some years earlier, Songer and Eatock reported non-quantal transmission [65], but their results did not explain the mechanism by which non-quantal transmission operates. Recently, Govindaraju et al. presented a computational model of what may be happening at this synapse [66]. However, the results of Contini et al. provide the definitive evidence of non-quantal transmission at this synapse and the mechanism by which it takes place. They showed that there are three factors governing transmission at this synapse:

- Glutamate release (quantal transmission);
- The level of potassium in the synaptic cleft;
- Resistive coupling between the type I receptor and the enveloping calyx (non-quantal transmission).

Each of these factors is addressed below:

(a) Glutamate release is apparently similar to that in the cochlea; vestibular type I receptors contain ribbon synapses, which release glutamate probably in response to relatively slow (sustained) stimuli. This is quantal glutamate transmission. The activation of the post-synaptic neuron by glutamatergic transmission is relatively slow [65].
(b) Vestibular stimulation causes the deflection of the stereocilia and therefore opens the mechanoelectrical transduction (MET) channels on the stereocilia of type I receptors, so potassium enters the type I receptor from the potassium-rich endolymph and is released by the receptor into the narrow (femtolitre) synaptic cleft between the type I receptor and its enveloping calyx. Potassium levels in the narrow synaptic cleft modulate the membrane potential of the receptor and the calyx [64,67,68].
(c) Most importantly, the simultaneous dual patch clamp recording of a type I receptor and its enveloping calyx afferent conclusively demonstrated non-quantal transmission experimentally. This is a form of synaptic transmission that is not dependent on glutamate reception on the post-synaptic membrane, and one key component of this non-quantal transmission is called resistive coupling, which is essentially electrical transmission that is dependent on membrane potential. This is a form of ultrafast electrical coupling between the type I receptor and calyx afferent; channels on the type I receptor membrane and the facing membrane of its calyx afferent are both open near resting potential, enabling the ultrafast depolarization of the irregular primary afferent [63,66]. We contend that this ultrafast non-quantal resistive coupling explains the precision of vestibular phase locking, the very short latency of the vCAP in comparison to the cCAP, and the extremely short latency of some irregular primary afferents to ACS clicks (0.5 ms) [69], and so we think resistive coupling is likely the key neural event in the generation of vCAP and therefore the generation of VEMPs

One consequence of blocking glutamate transmission via CNQX is that after CNQX blockade, a stimulus will still open the MET channels on the stereocilia of the type I receptors and will still cause receptor membrane depolarization (and therefore vestibular microphonics). Receptor membrane depolarization enables ultrafast resistive coupling between the type I receptor and its calyx to take place independently of the (relatively slow) quantal glutamatergic synaptic transmission [65,70]. This explains why the vestibular microphonic and the vCAP will be relatively unaffected by CNQX’s blockade of glutamatergic transmission.

### 3.5. Temporal Precision of Irregular Vestibular Afferents—Latency

**Figure 5:**
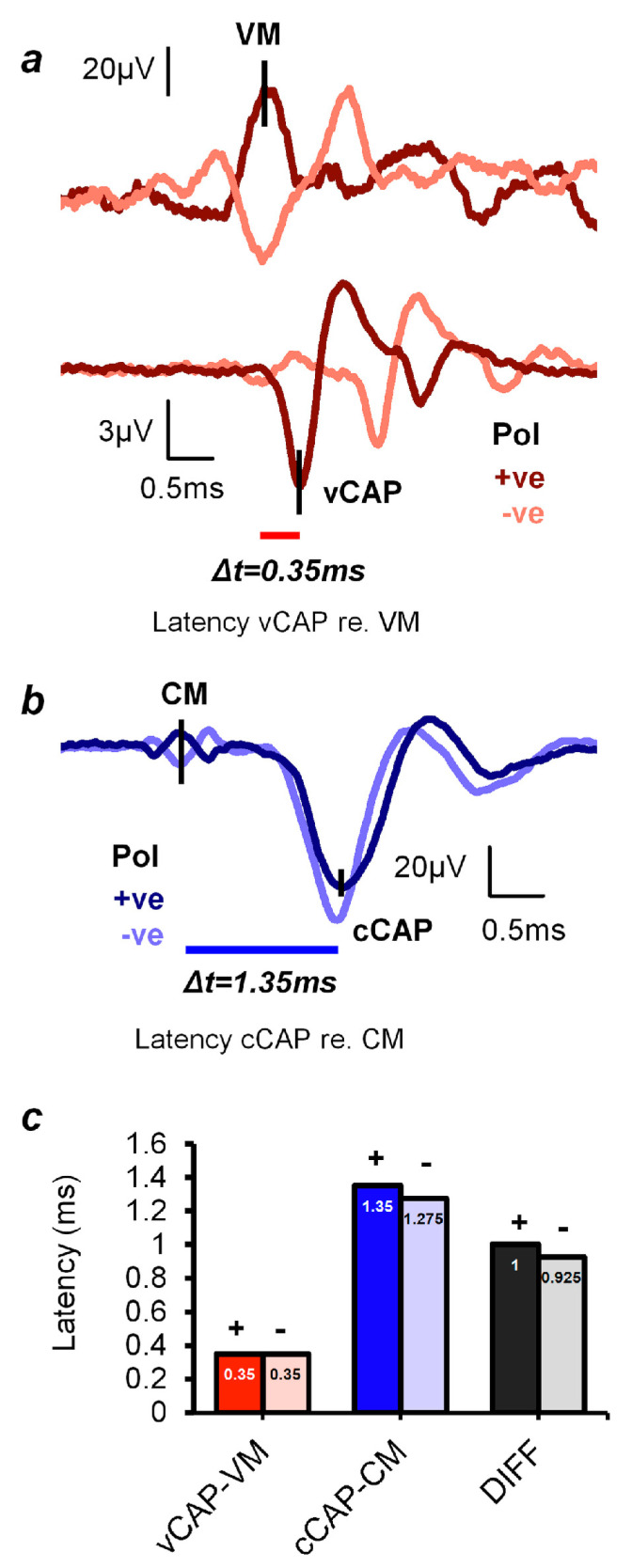
vCAP and cCAP response latencies, according to the peaks of the respective microphonic responses (CM and VM) to click stimuli. (**a**,**b**) Simultaneous recordings of the VM and vCAP and the CM and cCAP from a representative animal taken from the surface of the utricular macula and the facial nerve canal, respectively. (**a**) The latency difference between the onset of the VM and the onset of the vCAP is 0.35 ms. (**b**) The latency difference between the onset of the CM and the onset of the cCAP is 1.35 ms. So, the cochlear afferent response to clicks is around 1 ms slower than the vestibular afferent response. (**c**) The averaged latencies and latency differences of the vCAP and cCAP (across five animals). Both vestibular and cochlear responses in (**a**) and (**b**) were evoked by an identical 0.6 ms air-conducted sound (ACS) pulse, with a 0.3 ms rise–fall time at a stimulus intensity 20 dB above the threshold. (The polarity of the stimuli is given by: +ve refers to positive polarity, and -ve refers to negative polarity). Reproduced with permission from the authors of [14].

By using the peaks of the cochlear and vestibular microphonics as the benchmark for comparing the latencies of the vCAP and the cCAP, we have shown that the vCAP has a shorter latency than the cCAP (see Figure 5). This result corresponds to the short latency of primary vestibular afferents to ACS clicks, with latencies of some primary vestibular afferents to air-conducted clicks being as short as 0.5 ms [69].

### 3.6. Temporal Precision of Irregular Vestibular Afferents—Phase Locking

**Figure 6:**
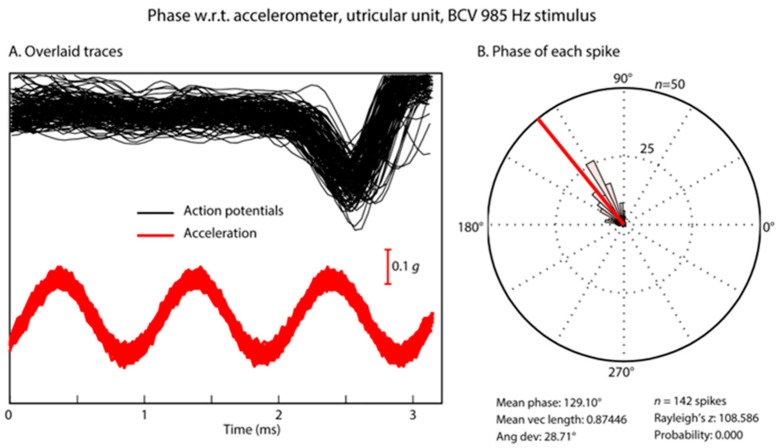
Phase locking of an irregular otolithic neuron to a sinusoidal BCV stimulus of 985 Hz BCV. (**A**) Time series of 142 action potentials in one neuron in response to the BCV stimulus (985 Hz) (shown by the red acceleration trace). Traces that contain an action potential are aligned using the timing of the stimulus pulse. (**B**) Circular histogram (rose plot) of the phase of each spike. The Rayleigh test of circular uniformity was performed on the 142 spikes, and the result was significant (*p* < 0.001), showing that the time when an afferent is activated is tightly phase locked to the stimulus. Here, the neuron misses many cycles, as can be seen from the value of the action potentials which contain no spikes in the cycles preceding each instance of firing, (**A**) At the moment when the neuron fires, it is locked to a narrow band of phase angles of the stimulus (**B**). Clearly, each individual cycle of the stimulus is acting to activate the receptor/afferent. Reproduced with permission from the authors of [1].

**Figure 7:**
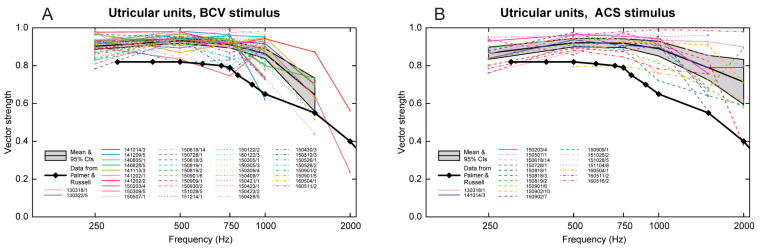
Precision of phase locking. Plots of vector strength (a measure of the strength of phase locking) versus frequency for BCV (panel (**A**)) and ACS (panel (**B**)) stimuli in guinea pig irregular utricular neurons. Data for different units are shown using different colors and line styles; the legend shows the date and number of each unit. The gray bands show the mean and 95% confidence intervals of the mean of all utricular units. The heavy black line with diamond symbols shows the estimated mean vector strength of guinea pig primary auditory neurons to sound (calculated from Figure 6 in [37]). Utricular neurons show similar high vector strengths for BCV and ACS, but the vector strengths of the utricular neurons are significantly higher than those of the primary auditory neurons in guinea pigs and extend to higher frequencies. Reproduced with permission from the authors of [53].

In response to sinusoidal stimuli, individual cochlear and vestibular afferents with irregular resting activity show precise phase locking; action potentials are generated at a particular phase angle (or a narrow band of phase angles) of the stimulus with high precision [24,35,53,71]. This does not mean that the neuron fires on every cycle of the stimulus; indeed, it may miss many cycles, but the moment when the neuron fires, it is locked to a particular phase angle of the sinusoidal stimulus [24] (see Figure 6). Phase locking in vestibular afferents is similar to that shown for cochlear afferent neurons; however, the precision of phase locking in irregular primary vestibular afferents to sound and vibration, as measured by vector strength, is as least as good as (and apparently superior to) that reported for cochlear afferents [37,53] (see Figure 7).

Further evidence of the temporal precision of irregular vestibular afferents can be found by examining the frequency limits of phase locking (Figure 7). The upper frequency of phase locking and average vector strength of vestibular primary afferents in the anesthetized guinea pig appears to be higher (3000 Hz) than that reported for cochlear primary afferents in [37] (Figure 7). We suggest that precise vestibular phase locking at high frequencies is most likely primarily due to resistive coupling, whereas recent evidence has shown that for cochlear afferents, the high precision of phase locking is most likely due to multiple ribbon synapses [24,34,72]. Tight vestibular phase locking may be due to the different mechanisms in the cochlear and vestibular systems.

## 4. Applications of Physiological Results to Clinical Vestibular (VEMP) Testing

In light of the above evidence, we consider how the cochlear and vestibular systems respond to various stimulus manipulations of relevance for clinical vestibular testing. In some experiments, these manipulations are carried out using the kind of procedures outlined above to eliminate or minimize cochlear contributions to the measured vestibular response.

### 4.1. Effect of Rise Time

The importance of stimulus onset in VEMP testing on human subjects was shown in a study in which the total duration of a 500 Hz tone burst stimulus was systematically decreased from 10 ms to 2 ms, leading to the oVEMP n10 for a 2 ms stimulus having the same amplitude as the response amplitude to a stimulus of 10 ms duration [73]. We have confirmed this result, which shows that it is the onset of the stimulus that is the key parameter in the generation of VEMPs.

**Figure 8:**
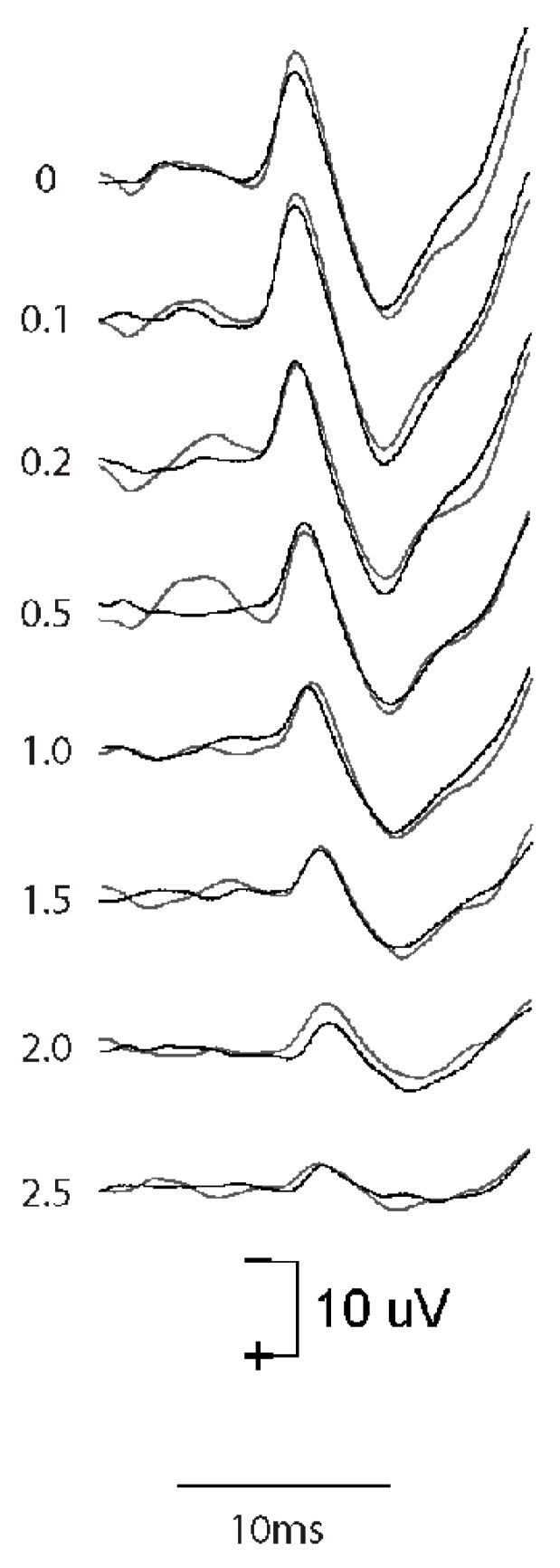
The effect of increasing rise time on the oVEMP n10 in one healthy subject. Increasing the rise time of the 500 Hz stimulus reduces the amplitude of the oVEMP n10 [6]. The number next to each record shows the rise time in milliseconds. Reproduced with permission from *Frontiers* [74].

The systematic variation in the rise time of short 500 Hz BCV tone bursts shows the importance of stimulus rise time in generating VEMPs in human subjects; increasing the rise time of 7 ms 500 Hz tone bursts decreased VEMP amplitude [6], with the largest VEMPs being to stimuli with a rise time of zero ms (see Figure 8).

**Figure 9:**
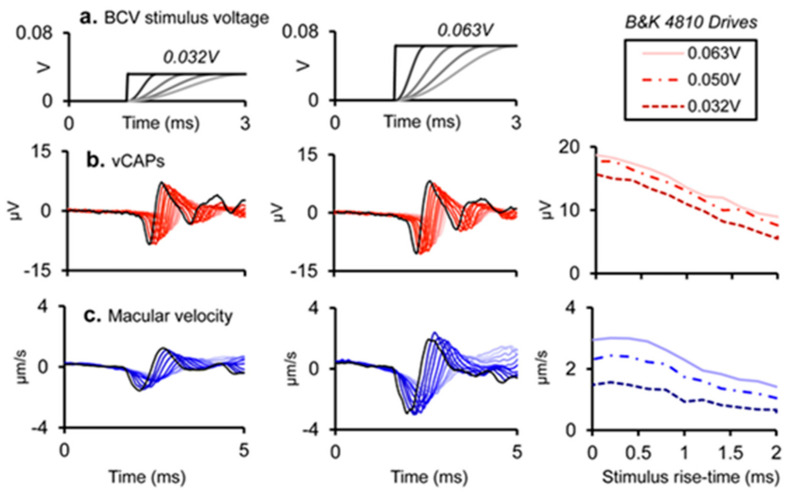
Simultaneous recordings of vCAPs and macular velocity during BCV stimulation as rise time is varied at two different intensities: columns 1 and 2 (drive voltages of 0.032 and 0.063 V). (**a**) The stimulus rise–fall time was varied between 0 and 2 ms for a 4 ms BCV monophasic pulse for 0.032 V (**Left panel**) and 0.063 V (**Middle panel**). Simultaneously measured responses include (**b**) vestibular compound action potentials (vCAPs) (red). (**c**) Laser Doppler vibrometry (LDV) measurements of utricular macular velocity recorded from a reflective microbead from the basal epithelial surface. Traces were superimposed as rise time was varied. Reproduced with permission from the authors of [14].

These clinical data correspond to the results of the primary vestibular afferents. We have shown that as the rise time of a click increases, the amplitude of the vCAP decreases. This is expected because increasing rise time will decrease the synchronization of action potentials across neurons [15] (see Figure 8). The optimum stimulus for maximum vCAPs (and therefore large VEMPs) is one with zero rise time [6,14] (see Figure 9).

Evidence from single neuron recordings shows the excellent temporal precision of irregular vestibular afferents, which respond with short latencies to transient stimuli [75] and phase lock with small variability to high frequencies [53]. However, some clinical studies continue to use long (2 ms or even longer) rise times for VEMP testing. Such stimuli generate modest but inferior VEMPs compared to those derived from using optimum stimuli. The use of such long rise times for VEMP testing is an inappropriate carryover from audiological testing of perceptual thresholds to pure tone stimuli, where it is necessary to use a long rise time in order to eliminate audible clicks at stimulus onset. However, in VEMP testing, the situation is exactly opposite to that in the audiometric testing of pure tone thresholds: for VEMPs, the most effective VEMP stimulus is one with a very short rise time, which synchronizes vestibular action potentials in primary afferent neurons. Unfortunately, some devices intended for use in VEMP testing do not allow for rise times less than 2 ms, and some clinicians do not realize how important it is to minimize the rise time for VEMP testing as opposed to auditory threshold testing.

### 4.2. Masking

The amplitude of the cCAP to ACS or BCV is reduced by masking (both simultaneous broadband masking or forward masking [59]), whereas the amplitude of the vCAP is minimally affected by masking. On the other hand, we have confirmed that in guinea pigs, vCAPs to ACS or BCV click stimuli are minimally affected by simultaneous broadband noise or forward masking by noise stimuli at levels which effectively mask cCAPs [20].

### 4.3. Paired Pulse Stimuli

**Figure 10:**
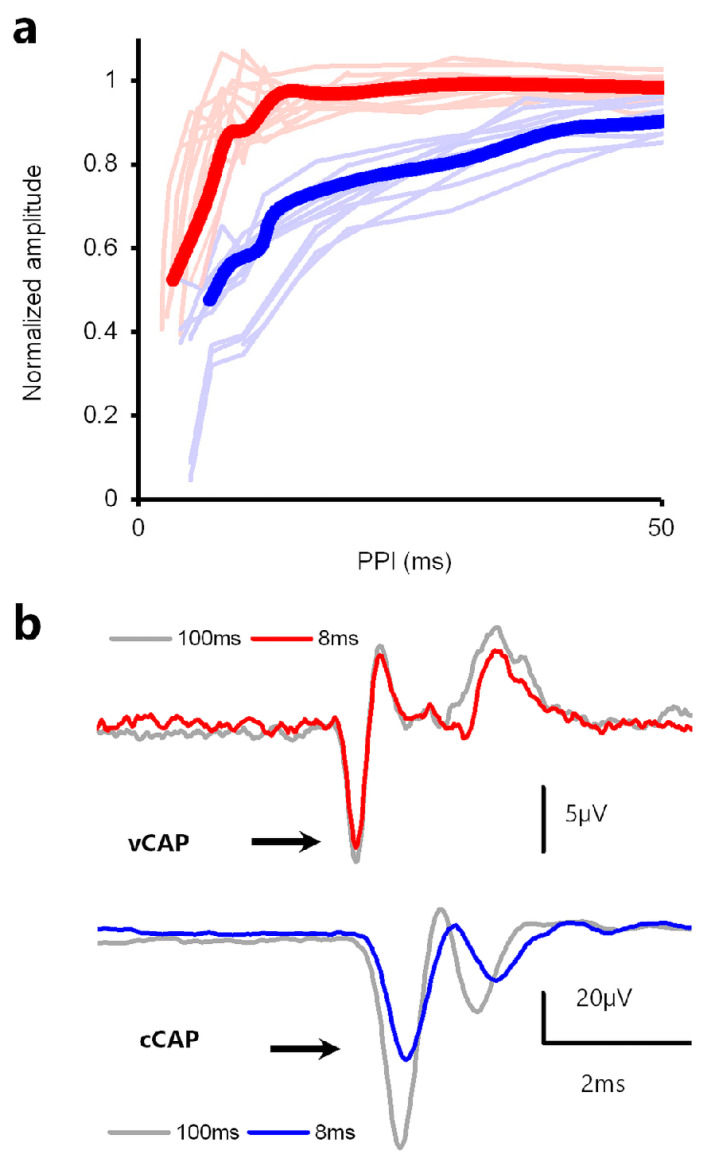
Forward masking of vCAPs vs. cCAPs in a paired-pulse paradigm. (**a**) Normalized amplitude of cCAPs evoked by paired-pulse stimuli (blue) vs. BCV-evoked paired-pulse vCAPs (red). (**b**) Example records showing lack of forward masking in vCAPs and significant forward masking in cCAPs for a paired pulse interval of 8 ms. Averaged vCAPs were insensitive to changes in PPI between 107 ms and 10 ms yet decreased with PPIs below ~8 ms. The VM amplitude was insensitive to changes across all PPIs. In contrast, the cCAP shows a substantial reduction in amplitude for cCAPs at 8 ms intervals. Reproduced with permission from the authors of [14].

Reducing the interval between successive pulses reduces the cCAP to the second pulse, with reductions in cCAP amplitude beginning at an interstimulus interval of about 50 ms and reaching a 50% response suppression at an interstimulus interval of ~10 ms [76,77] (see Figure 10). However, in the same paradigm, the vCAP to the second pulse is minimally reduced at short interstimulus intervals [59] (see Figure 10).

Vestibular afferents are resistant to masking, whereas with cochlea afferents, it is relatively easy to mask their response. We contend that the differences in synaptic transmission (cochlea vs. vestibular) are responsible for this differential effect regarding stimuli masking.

## 5. Other Stimuli

For both auditory and cochlear systems, there are other components and variants of these labyrinthine electrophysiological potentials to stimuli other than transients (see [16]), including those listed below, but these are beyond the scope of this review.

1. The vestibular evoked potential at the scalp (VsEP);
2. The vestibular summating potential [17];
3. Auditory Nerve Neurophonic (ANN) [78];
4. Vestibular Nerve Neurophonic (VNN).

## 6. Conclusions

One outcome of measuring vCAPs is that they allow us to evaluate which particular stimulus parameters at the level of otolithic receptors are likely optimal for clinical VEMP testing. So, for example, the results have confirmed the importance of short rise times in optimizing VEMP stimuli. A simple way of reducing cochlear contributions to vestibular responses is to use simultaneous masking noise, as confirmed by the physiological results presented in this paper. Another preliminary result is that the CE chirp stimulus (500–4000 Hz sweeps), also called narrow-band chirps, are particularly clinically effective stimuli for oVEMPs [79,80]. Our recordings of the vCAP in response to chirp stimuli confirm that the chirp stimulus with a rise time of zero is particularly effective in generating vCAPs at the level of primary afferents in comparison to simple clicks [12].

## 7. Summary

1. It is otolithic receptors and their afferents with irregular resting discharge originating from the striola which are activated by sound and vibration (for reviews, see [4,49,54] and therefore responsible for the vCAP.
2. Vestibular receptors and afferents can function independently of the cochlea, both in humans and guinea pigs.
3. It is possible to differentiate between the vestibular and cochlear responses to transient stimuli. Such differentiation provides further support to the basis of present vestibular testing, which usually involves using sound and vibration stimuli, and may allow new clinical tests of dynamic vestibular function. For future studies of putative vestibular responses to clinically realistic transient stimuli, it is advisable to have continuous broadband masking present simultaneously to minimize cochlear contributions in the response(s) [27].
4. In clinical VEMP testing, the situation is exactly opposite to that in the audiometric testing of pure tone thresholds; effective VEMP stimuli have a very short rise time, synchronizing vestibular action potentials in primary afferent neurons. In contrast, for the audiometric testing of thresholds, a long rise time is mandatory. Unfortunately, some stimulus generators do not allow for rise times less than 2 ms, and some clinicians who measure VEMPs do not realize how important it is to minimize the rise time for VEMP testing as opposed to auditory threshold testing. Our recordings of the vCAP in response to chirp stimuli confirm that the chirp stimulus is particularly effective in generating vCAPs at the level of primary afferents in comparison to simple clicks.

## Data Availability

Not applicable.

## Dedication

This paper is a tribute to Wally Grant—a great friend and a brilliant scientist and engineer whose insights and contributions advanced the understanding of so many areas of vestibular function. Wally Grant died on 12 July 2023.

## Abbreviations

CAP	compound action potential
vCAP	vestibular compound action potential
cCAP	cochlear compound action potential
CM	cochlear microphonic
VM	vestibular microphonic
ANN	auditory nerve neurophonic
VNN	vestibular nerve neurophonic
ACS	air-conducted sound
BCV	bone-conducted vibration
SCD	semicircular canal dehiscence
VEMP	vestibular evoked myogenic potential
VsEP	vestibular evoked potential
oVEMP	ocular vestibular evoked myogenic potential
CNQX	6-cyano-7-nitroquinoxaline-2,3-dione
MET	mechanoelectrical transduction
KCl	potassium chloride
PPI	paired pulse interval
LDV	laser Doppler vibrometer
